# The role of adjuvant chemotherapy in the management of acute promyelocytic leukemia differentiation syndrome

**DOI:** 10.3389/fonc.2022.911745

**Published:** 2022-08-03

**Authors:** Dominic LaBella, Samuel Regan, Heiko Konig, Daniel N. Egan, Neil A. Bailey, Raya Mawad, Morgan Gilbert, John M. Pagel, Jeffrey J. Pu

**Affiliations:** ^1^ Department of Medicine, State University of New York Upstate Medical University, Syracuse, NY, United States; ^2^ Department of Radiation Oncology, Duke University, Durham, NC, United States; ^3^ Department of Medicine, Indiana University School of Medicine, Indianapolis, IN, United States; ^4^ Department of Medicine, Swedish Medical Center, Seattle, WA, United States; ^5^ Department of Medicine, University of Arizona National Cancer Institute (NCI) Designated Comprehensive Cancer Center, Tucson, AZ, United States

**Keywords:** Acute Promyelocytic Leukemia, All-Trans-Retinoic Acid (ATRA), Arsenic Trioxide (ATO), Differentiation Syndrome (DS), Adjuvant Chemotherapy

## Abstract

Acute Promyelocytic Leukemia (APL) is characterized by the t(15;17) chromosomal translocation resulting in a PML-RARA fusion protein. The all-trans-retinoic acid (ATRA) and Arsenic Trioxide (ATO) only regimens have demonstrated success in treating low- and intermediate-risk patients. However, induction with ATRA/ATO only regimens have been showing increased incidence of differentiation syndrome (DS), a potentially lethal complication, traditionally treated with dexamethasone. We conducted a three-institution retrospective study, aiming to evaluate the role of short-term adjuvant chemotherapy in managing moderate DS for patients with low- or intermediate-risk APL initially treated with ATRA/ATO only protocols. We evaluated the difference in incidence and duration of moderate DS in APL patients who were treated with ATRA/ATO with or without adjuvant chemotherapy. 57 low- or intermediate-risk APL patients were retrospectively identified and included for this study; 36 patients received ATRA/ATO only induction treatment, and 21 patients received ATRA/ATO/adjuvant chemotherapy combination induction therapy. Similar proportions of patients experienced DS in both groups (66.7% vs. 81.0%, P = 0.246). The median duration of DS resolution in patients receiving ATRA/ATO only was 17 days (n = 23), and in patients receiving combination therapy was 8 days (n = 16) (P = 0.0001). The lengths of hospital stay in patients receiving ATRO/ATO only was 38 days (n = 7), and in patients receiving combination therapy was 14 days (n = 17) (P = 0.0007). In conclusion, adding adjuvant chemotherapy to ATRA/ATO only protocol may reduce the duration of DS and the length of hospital stay during APL induction treatment.

## Introduction

Acute Promyelocytic Leukemia (APL) is characterized by the t(15;17) chromosomal translocation resulting in a PML-RARA fusion protein. APL is stratified into low-, intermediate-, and high-risk subgroups based on pre-treatment white blood cell (WBC) and platelet counts. Traditional APL-directed therapy typically consists of anthracycline/cytarabine-based chemotherapy regimens. However, high relapse rates, considerable treatment-related toxicity, and therapy-induced neoplasms remained a concern for this approach (1). Since the discovery of the unique effects of all-trans-retinoic acid (ATRA) and Arsenic Trioxide (ATO) on APL cell differentiations, these agents have been incorporated into APL treatment protocols and lead to significant improvements in treatment outcome. Two large phase III clinical trials, AML17 and APL0406, evaluated the role of ATRA/ATO only therapy compared to combination with traditional chemotherapy regimens and demonstrated that ATRA/ATO only regimens improve event free survival with acceptable toxicities, particularly in low and intermediate risk patients (1, 2).

However, induction with ATRA/ATO only regimens have been showing increased incidence of differentiation syndrome (DS), a potentially lethal complication (3, 4). In many clinical scenarios, the supportive measures were not able to sufficiently control DS (4, 5). For these patients, a short-term adjuvant cytotoxic chemotherapy may be required to mitigate DS symptoms. However, the role, timing, and impact of short-term adjuvant chemotherapy for DS management were not investigated in the AML17 and APL0406 trials. Moderate DS is defined as having two or three of the following signs and symptoms: dyspnea, unexplained fever, weight gain greater than 5kg, unexplained hypotension, acute renal failure, and a chest radiograph demonstrating pulmonary infiltrates of pleuropericardial effusion (3).

We conducted a three-institution retrospective real-world practice data study aimed to evaluate the role of short-term adjuvant chemotherapy in managing moderate DS for patients with low- or intermediate-risk APL initially treated with ATRA/ATO only protocols.

## Methods

This study was IRB-approved and retrospectively recruited patients diagnosed with low- or intermediate-risk APL and treated with ATRO/ATO protocol between July 1, 2016 and January 21, 2022. The patients were retrospectively grouped into 2 cohorts (A, B): cohort A received ATRA/ATO only therapy, and cohort B received a short-term anthracycline/or cytarabine–based chemotherapy in addition to ATRA/ATO therapy for the purpose of treatment of moderate DS. To objectively evaluate the onset and resolution of DS, Frankel et al’s definition of moderate DS was utilized (3). Data collection and statistical analysis were conducted using RedCap and Microsoft Excel. A one-tailed z-score test for two patient groups was performed to determine the difference in incidence of DS among patients who receded or didn’t receive the adjuvant chemotherapy treatments, the difference in proportion of patients dying from DS, and the difference in APL response rates. A Kruskal-Wallis test was performed to determine the days to DS from ATRA/ATO induction and the difference in DS durations between the patients in cohort A and cohort B. A Pearson Correlation test was performed to determine significance of a linear correlation between initial WBC and duration of DS for adjuvant chemotherapy and ATRA/ATO groups independently.

## Results and discussion

**Figure 1:**
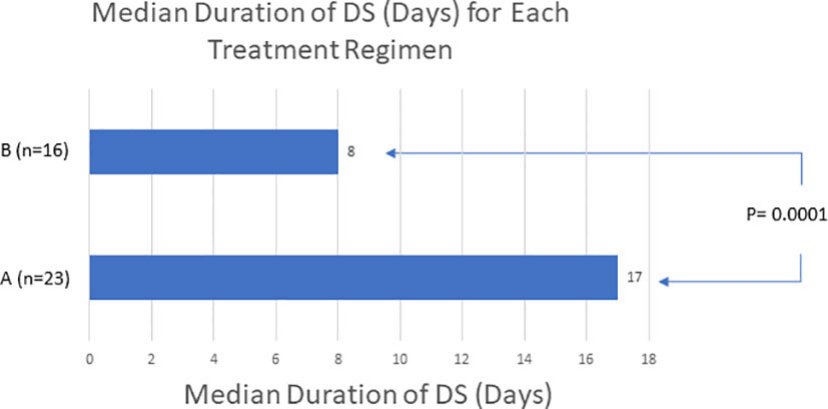
The use of adjuvant chemotherapy to patients on ATRA/ATO only protocols may reduce the duration of DS. ‘A’ refers to patients receiving ATRA/ATO only therapy. ‘B’ refers to patients receiving both ATRA/ATO therapy and adjuvant chemotherapy. This data demonstrates a shorter duration of DS with adding adjuvant chemotherapy.

57 low- or intermediate-risk APL patients were retrospectively identified and included for this study, 36 patients in cohort A and 21 patients in cohort B (**Table 1**). The APL diagnosis was confirmed by employing FISH analysis to detect t(15;17) translocation of PML-RARA fusion gene. There were 29 male and 28 female patients included in the study. The median WBC count at diagnosis was 1.8 x 10^9/^liter [range 0.3 x 10 (9) to 7.1 x 10 (9)]. At the time of diagnosis, 47 and 10 patients were in the low- and intermediate-risk APL group respectively. DS developed in 41 (71.9%) patients. In total, 24 of 36 (66.7%) patients in cohort A and 17 of 21 (81.0%) patients in cohort B developed DS (P = 0.246). The median time to DS from initiation of ATRA/ATO induction therapy in cohort A was 5 days (range, 0 to 26, n = 24), and the median time to DS from initiation of ATRA/ATO induction therapy in cohort B was 3 days (range, 2 to 15, n = 17) (P = 0.037). For the 17 patients in cohort B that developed DS, 4 were administered adjuvant chemotherapy 1 day before showing laboratory evidence of DS, 8 were after the onset of DS, and 5 were on the same day as DS onset. Twenty-three of 24 patients who experienced DS in cohort A eventually had their DS resolved. The 1 patient without DS resolution in cohort A died 11 days after DS began. Similarly, 16 of the 17 patients who experienced DS in cohort B had their DS resolved; 7 of these patients were treated with idarubicin, 8 of these patients were treated with daunorubicin, and 1 of these patients were treated with cytarabine. The 1 patient without DS resolution in cohort B died 8 days after DS began. The median duration of DS resolution in cohort A was 17 days (n = 23), and in cohort B was 8 days (n = 16) (P = 0.0001) (**Figure 1**). All patients that developed DS were treated with dexamethasone regimens throughout the duration of DS as well as stopping the offending induction agents. The median hospitalization duration was 38 days in cohort A patients (n = 7), and 14 days in cohort B patients (n = 17) (P = 0.0007). The proportion of patients who survived from DS was 23 of 24 (95.8%) in cohort A, and 16 of 17 (94.1%) in cohort B (P = 0.803). 35 of the 36 patients in cohort A had their APL treatment response data available in chart review, and all 18 of the patients in cohort B had their APL treatment response data available in chart review. The proportion of patients who had APL complete response in cohort A was 32 of 35 (91.4%) and in cohort B was 18 of 21 (85.7%) (P = 0.503). Twenty-nine of the 32 patients who had an APL complete response in cohort A had a duration to APL complete response available in chart review, and all 18 of the 18 patients with complete response in cohort B had a duration to APL complete response available. The median days to APL complete response in cohort A was 40 days (n = 29), and cohort B was 33.5 days (n = 18) (P = 0.0941).

**Table 1:** Patient characteristics and outcomes.

Baseline characteristics	Cohort	B (n=21)
	A (n=36)	
Median age at diagnosis (years)	58.5	49
Gender (n)		
Male	18	11
Female	18	10
APL severity		
Low	33	14
Intermediate	3	7
Chemotherapy (n)		
Cytarabine	0	3
Daunorubicin	0	12*
Idarubicin	0	8**
Differentiation Syndrome		
Yes (P=0.246)	24	17
No	36	21
Median days to Differentiation Syndrome from ATRA/ATO induction		
Median (P=0.037)	5	3
n	24	17
Median Differentiation Syndrome Resolution Duration (days)		
Median (P=0.0001)	17	8
n	23	16
Hospital Course Duration		
Median (P=0.0007)	38	14
n	7	17
Proportion of Patient Survival from DS		
Survive (n) (P=0.803)	23	16
Death (n)	1	1
Percentage Survival (%)	95.8	94.1
APL Response		
Complete Response (n) (P=0.503)	32	18
Non-Complete Response (n)	3	3
Unknown (n)	1	0
Percentage Complete Response (%)	91.4	85.7
Days to Complete Response		
Median (P=0.0941)	40	33.5
n	29	18

*one patient was treated with cytarabine and idarubicin.

**one patient was treated with cytarabine and daunorubicin.

Since the implementation of ATRA and ATO for APL target therapy in the 1990s, APL treatment outcomes have significantly improved. Two phase III clinical trials, AML17 and APL0406, have demonstrated that ATRA and ATO without chemotherapy is efficacious, particularly in non-high-risk populations. The AML17 trial compared ATRA with ATO or ATRA with idarubicin and mitoxantrone. ATRA/ATO treatment resulted in a lower relapse rates and higher event free survival compared to the standard chemotherapy regimen, though no differences in overall survival or quality of life were noted (1). The APL0406 trial compared ATRA and ATO therapy with ATRA and chemotherapy in low- or intermediate-risk APL patients. The authors concluded that ATRA/ATO therapy is non-inferior to ATRA/chemotherapy regimens, with improved event free survival, disease free survival, overall survival, and acceptable toxicities (2).

Frequently reported acute toxicities of ATRA and ATO therapies include QTc prolongation, elevated liver enzymes, hyperleukocytosis, and DS (4). Hyperleukocytosis occurs during induction therapy with differentiating agents in up to 70% of non-high-risk patients (4). The reported incidence of DS varied due to diagnostic criteria discrepancies and differing induction regimens, and has ranged from 2% to 48% (5, 6). A retrospective study of 259 APL patients treated with ATRA and chemotherapy reported that 13.5% of patients experienced early death and 17.8% experienced DS, and progressive hyperleukocytosis during induction predicted both early death and DS (5).

**Figure 2:**
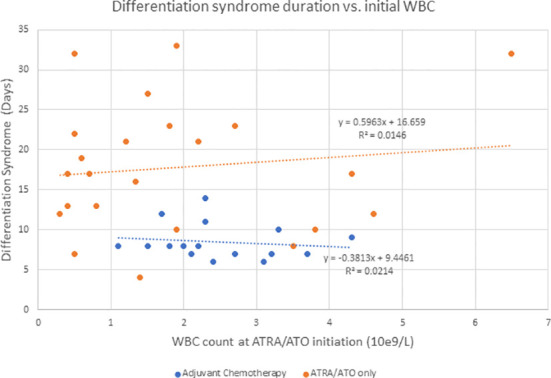
Describes the duration of DS when compared to initial WBC counts at the initiation of ATRA/ATO therapy for the adjuvant chemotherapy group and ATRA/ATO only group independently.

**Figure 3:**
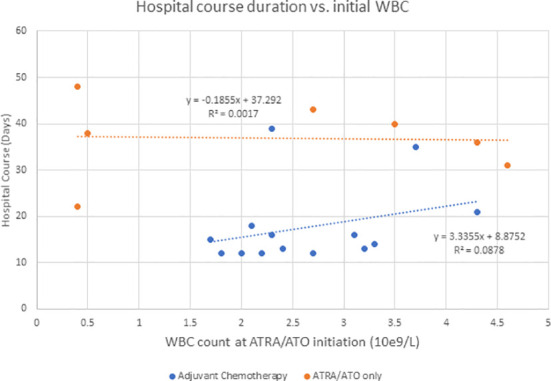
Describes the duration of hospitalization when compared to initial WBC counts at the initiation of ATRA/ATO therapy for the adjuvant chemotherapy group and ATRA/ATO only group independently.

Hyperleukocytosis and DS rates are higher in ATRA/ATO regimens compared to ATRA + chemotherapy regimens, likely because they do not result in significant cytotoxic cytoreduction (6). In addition to supportive measures, management of DS includes dose reduction or discontinuation, corticosteroids, anthracyclines, or hydroxyurea for leukoreduction (4–6). Ciftciler et al. describes how early death in APL can be attributed to a number of factors in addition to DS. They proposed that early death is most attributed to severe hemorrhage, which is due to coagulopathy disorders from low fibrinogen and initially high WBC counts (7). Our study showed that there was not a significant correlation between initial WBC and duration of DS for the adjuvant chemotherapy (P = 0.589) or the ATRA/ATO only group (P = 0.582) (**Figure 2**). Additionally, our study showed that there was not a significant correlation between initial WBC and duration of hospitalization for the adjuvant chemotherapy (P = 0.304) or the ATRA/ATO only group (P = 0.930) (**Figure 3**). In our study, the median days to complete remission (CR) was similar in those 2 cohorts (P = 0.0941), indicating non-inferiority of adding short-term adjuvant chemotherapy in time to complete response.

**Figure 4:**
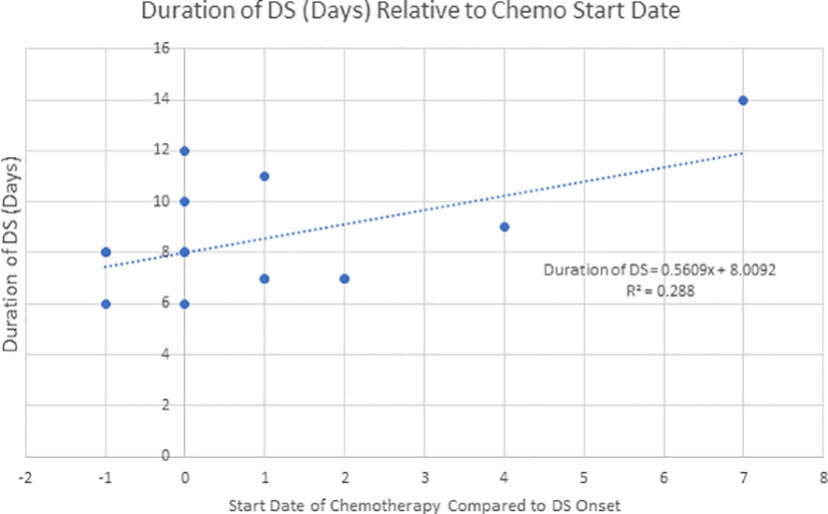
Describes the duration of DS when compared to the start date of adjuvant chemotherapy relative to the onset date of DS (n=16, R2 = 0.288). This data demonstrates a trend of earlier starting adjuvant chemotherapy and shorter DS duration.

In real world practice, adjuvant chemotherapy has been frequently used for DS management. In this study, both cohorts A and B contained a similar proportion of patients suffering from DS (P = 0.246), indicating their comparable biological responses to ATRA/ATO therapies. However, this study showed a significant trend towards a shorter DS duration when adding adjuvant chemotherapy to ATRA/ATO therapy (P = 0.0001), especially when used earlier in the onset of DS (**Figure 4**). We propose that early management of DS with adjuvant chemotherapy may result in earlier resolution and potentially improved patient outcomes and decreased healthcare-associated costs. Our study counted a similar 91.4% CR for cohort A and 85.7% for cohort B (P = 0.503).

There is a concern that chemotherapy would increase the risk of secondary neoplasms. In APL0406 and AML17 studies, only 2 and 1 therapy-related neoplasms were observed in the ATRA/chemotherapy arm, respectively (1, 2). Our study demonstrated that short-term use of adjuvant chemotherapy for DS management might bring more benefit than secondary cancer risk.

In conclusion, the use of short-term adjuvant chemotherapy to patients on ATRA/ATO only protocol may reduce the duration of DS. Short-term adjuvant chemotherapy intervention is currently underutilized and there is no guideline on how and when to use this treatment strategy in preventing or treating DS. Large-scale randomized prospective studies are warranted to further validate the conclusion from this retrospective study and to analyze the effects of ATRA/ATO and short-term adjuvant chemotherapy in high-risk APL patients.

## Data availability statement

The original contributions presented in the study are included in the article/**Supplementary Material**. Further inquiries can be directed to the corresponding author.

## Ethics statement

The studies involving human participants were reviewed and approved by Upstate Medical University. Written informed consent for participation was not required for this study in accordance with the national legislation and the institutional requirements.

## Author contributions

JJP initiated the research idea and concept. JJP, DL, and SR designed this study and formulated the manuscript. JJP, JMP, HK, DE, NB, RM, and MG provided patient care. All authors contributed to the article and approved the submitted version.

## Funding

This study was supported by, AA&MDSIF research grant to JJP (146818), American Cancer Society grant to JJP (124171-IRG-13-043-02), NIDA/FDA research grant to JJP (P50 DA036107), JTTai & Co Foundation Cancer Research Grant to JJP, Paige’s Butterfly cancer research grant, NIH/NCI grant to JJP (P30CA023074), and a SUNY Upstate Medical University research grant to JJP.

## Acknowledgments

The authors would like to thank patients and nurses for supporting us in advancing disease management knowledge and patient care experience.

## Conflict of interest

The authors declare that the research was conducted in the absence of any commercial or financial relationships that could be construed as a potential conflict of interest.

## Publisher’s note

All claims expressed in this article are solely those of the authors and do not necessarily represent those of their affiliated organizations, or those of the publisher, the editors and the reviewers. Any product that may be evaluated in this article, or claim that may be made by its manufacturer, is not guaranteed or endorsed by the publisher.

## References

[1] Burnett AK Russell NH Hills RK Bowen D Kell J Knapper S. Arsenic trioxide and all-trans retinoic acid treatment for acute promyelocytic leukaemia in all risk groups (AML17): Results of a randomised, controlled, phase 3 trial. Lancet Oncol (2015) 16(13):1295–305. doi: 10.1016/S1470-2045(15)00193-X 26384238

[2] Platzbecker U Avvisati G Cicconi L Thiede C Paoloni F Vignetti M. Improved outcomes with retinoic acid and arsenic trioxide compared with retinoic acid and chemotherapy in non-High-Risk acute promyelocytic leukemia: Final results of the randomized Italian-German APL0406 trial. J Clin Oncol (2017) 35(6):605–12. doi: 10.1200/JCO.2016.67.1982 27400939

[3] Montesinos P Bergua J Vellenga E Rayón C Parody R de la Serna J. Differentiation syndrome in patients with acute promyelocytic leukemia treated with all-trans retinoic acid and anthracycline chemotherapy: characteristic, outcome, and prognostic factos. Blood (2019) 113(4):775–83. doi: 10.1182/blood-2008-07-168617 18945964

[4] Sanz MA Fenaux P Tallman MS Estey EH Lowenberg B Naoe T. Management of acute promyelocytic leukemia: Updated recommendations from an expert panel of the European LeukemiaNet. Blood (2019) 133(15):1630–43. doi: 10.1182/blood-2019-01-894980 PMC6509567 30803991

[5] Yoon J-H Kim H-J Min GJ Park SS Jeon YW Lee SE. Progressive hyperleukocytosis is a relevant predictive marker for differentiation syndrome, early death, and subsequent relapse in acute promyelocytic leukemia. Sci Rep (2019) 9(1):11935. doi: 10.1038/s41598-019-47937-4 31417123 PMC6695497

[6] Stahl M Tallman MS. Differentiation syndrome in acute promyelocytic leukaemia. Br J Haematol (2019) 187(2):157–62. doi: 10.1111/bjh.16151 31410848

[7] Ciftciler R Haznedaroglu I Aksu S Ozcebe O Sayınalp N Malkan UY. The factors affecting early death in newly diagnosed APL patients. Open Med (2019) 14(1):647–52. doi: 10.1515/med-2019-0074 PMC6744608 31565673

